# Association of inflammation and abnormal lipid metabolism with risk of thrombosis and thrombosis progression in patients with polycythemia vera: a retrospective study

**DOI:** 10.1007/s00277-023-05518-6

**Published:** 2023-11-01

**Authors:** Hurong Lai, Yansong Tu, Shan Zhang, Caifeng Liao, Huaijun Tu, Jian Li

**Affiliations:** 1 The Key Laboratory of Hematology of Jiangxi Province, The Department of Hematology, The Second Affiliated Hospital of Nanchang University, 1 Minde Road, Nanchang, 330006 Jiangxi China; 2 Graduate School of Medicine, Nanchang University, 465 Bayi Road, Nanchang, 330006 Jiangxi China; 3 Faculty of Science, University of Melbourne Grattan Street, Parkville, VIC 3010 Australia; 4 The Department of Geratology, The Second Affiliated Hospital of Nanchang University, 1 Minde Road, Nanchang, 330006 Jiangxi China

**Keywords:** Polycythemia vera, Inflammatory, Abnormal lipid metabolism, Thrombosis progression, Retrospective study

## Abstract

To date, no therapeutic strategy has been shown to be effective in reducing the risk of polycythemia vera (PV) transforming into myelofibrosis or leukemia, and the main goal of current treatment is to prevent thrombotic events. Recent studies have shown that higher levels of inflammation are associated with an increased risk of thrombosis in PV patients, while the correlation between inflammation and abnormal lipid metabolism with the risk of thrombosis in PV has not been reported. In this retrospective study, 148 patients with newly diagnosed PV who visited the Affiliated Hospitals of Nanchang University from January 2013 to June 2023 were categorized into low-risk group and high-risk group according to the risk of thrombosis, and were subsequently divided into thrombosis non-progression group and progression group. The differences of novel inflammatory markers PHR, NHR, MHR, LHR, and SIRI in each group were analyzed and compared with healthy adults who underwent physical examination in the hospitals during the same period. The results showed that PHR, NHR, MHR, and SIRI levels were significantly higher in the PV group than in the control group (*P* < 0.001), while HDL-C levels were considerably lower (1.09 vs. 1.31, *P* < 0.001). Comparisons within the groups of PV patients revealed that PHR, MHR, NHR, NLR, and SIRI levels were significantly higher in the high-risk group for thrombosis than in the low-risk group (*P* < 0.01); the thrombosis PHR, NHR, NLR, and SIRI levels were higher in the group with progression of thrombosis than in the group without progression of thrombosis (*P* < 0.05), while HDL-C levels were significantly lower (1.02 vs. 1.12, *P* < 0.001). The results of the ROC curve analysis showed that NHR (AUC = 0.791), HDL-C (AUC = 0.691), PHR (AUC = 0.668), NLR(AUC = 0.658), and SIRI (AUC = 0.638) had high diagnostic efficacy for identifying PV patients with thrombosis progression. Multivariate analysis showed that NHR, NLR, MHR, and LHR were independent risk factors for PV patients with thrombosis progression (*P* < 0.05). Kaplan–Meier survival curves showed that NHR ≥ 5.82 × 10^9^/mmol, NLR ≥ 6.295, PHR ≥ 280.4 × 10^9^/mmol, MHR ≥ 0.295 × 10^9^/mmol, LHR ≥ 1.41 × 10^9^/mmol, and SIRI ≥ 1.53 × 10^9^/L were risk factors for PFS in PV patients. The study demonstrates for the first time that novel inflammatory markers PHR, NHR, MHR, LHR, and SIRI may be used as new predictors for PV patients with thrombosis progression. NHR has the highest value in predicting thrombosis in PV patients and is superior to NLR which was reported previously.

## Introduction

Polycythemia vera (PV) is the prevalent subtype of BCR-ABL1 negative myeloproliferative neoplasms (MPN), with an incidence of 1.09/100,000 and a median survival time of 13.7 years [1, 2]. Thromboembolism is the most common complication of PV. The results of large clinical studies have shown that the incidence of thromboembolism in PV patients is about 46%, while the incidence of arterial thromboembolism is two to three times higher than that of venous thromboembolism [3, 4]. In recent years, the incidence of PV-related thromboembolism has been increasing year by year, and the comprehensive management of PV thromboembolism has ushered in a new therapeutic challenge.

Recent studies have suggested that inflammation plays a significant role in PV patients with thrombosis. The inflammatory marker neutrophil-to-lymphocyte ratio (NLR) can be used as an independent risk factor and a new predictor for PV patients with thrombosis [5, 6]. High-density lipoprotein (HDL) has anti-inflammatory, anti-oxidation, and anti-platelet aggregation properties and thus may play an antithrombotic role in cardiovascular and cerebrovascular diseases. The results of a cohort study based on 500 participants confirmed that higher levels of HDL were associated with a reduced risk of lower extremity deep venous thrombosis (LEDVT) [7]. We all know that statins could increase the expression level of HDL in serum. Podoltsev et al. had found that statins could reduce the risk of thrombosis in PV patients, so HDL had a potential association with risk of thrombosis in PV patients [8]. New hematological parameters which are made of HDL and complete blood count, such as platelet/HDL ratio (PHR), neutrophil/HDL ratio (NHR), monocyte/HDL ratio (MHR), lymphocyte/HDL ratio (LHR), and systemic inflammatory response index (SIRI), have been proposed as new inflammatory biomarkers. These parameters are also significantly related to the severity and poor prognosis of elderly acute myocardial infarction, multiple Myeloma, Parkinson’s disease, metabolic syndrome, acute coronary syndrome, and other diseases [9–14]. In the last few years, with the description of JAK2, MPL and CALR driver mutations, scores which were used to identify PV patients with different thrombotic risks have improved and largely guided treatment decisions [15]. Existing PV thrombosis risk groupings are categorized into low-risk group and high-risk group based on age and previous PV-related thrombosis, but still lack early predictors for thrombosis progression during follow-up [16]. Currently, no studies have been reported on the correlation between inflammation and abnormal lipid metabolism in PV patients with thrombosis progression during follow-up. Based on the bidirectional effects of serum inflammatory markers and HDL on thrombosis, the research has firstly explored the correlation of PHR, NHR, MHR, LHR, and SIRI with thrombosis risk and thrombosis progression during follow-up in PV patients. It is expected to provide cheaper and simpler reference indicators for early clinical prediction of thrombosis risk and thrombosis progression in PV patients.

## Patients and methods

### *Patients*

**Fig. 1:**
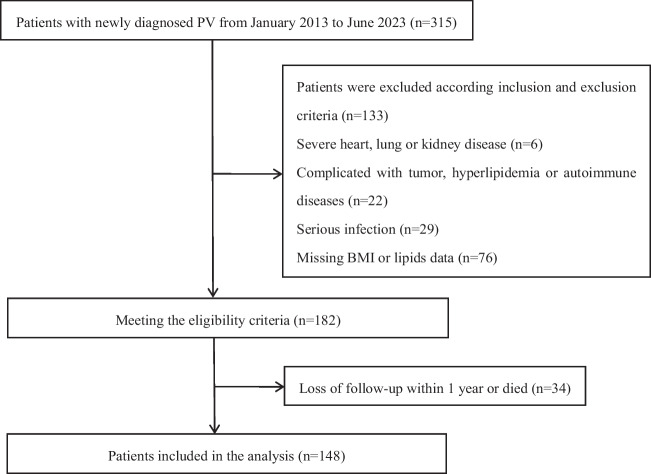
Patients selection flow chart

A total of 148 newly diagnosed PV patients in the Second Affiliated Hospital of Nanchang University, the First Affiliated Hospital of Nanchang University and Jiangxi Provincial People’s Hospital from January 2013 to June 2023 were enrolled in this retrospective study. All the patients were tested for JAK2 mutation gene and BCR-ABL fusion gene and were retrospectively diagnosed according to the 2016 WHO PV diagnostic criteria [17]. The median follow-up time of PV patients was 48.25 (23.18–68.5) months. The inclusion and exclusion criteria are shown in Table 1, and the patient selection flow chart is shown in Fig. 1. All PV patients were divided into low-risk group and high-risk group according to the risk of thrombosis and were categorized into thrombosis non-progression group and thrombosis progression group according to whether the progression of thrombosis has occurred. In order to compare the differences in serum inflammatory markers and HDL levels between PV patients and healthy adults, 148 healthy adults who underwent physical examination in our hospital were also included as a control group and then matched for age and sex with PV patients. The major participating center of study is the Second Affiliated Hospital of Nanchang University. The study has been approved by the Institutional Ethics Committee of the hospital. In addition, informed consents were obtained from all individual participants included in the study.

**Table 1:** Inclusion and exclusion criteria

Inclusion criteria	Exclusion criteria
a) Gender and age were not limited b) Conform to the diagnostic criteria of PV in《the 2016 revision to the World Health Organization classification of myeloid neoplasms and acute leukemia》^[[16]]^ c) Data on JAK2 mutations, complete blood counts and lipid profiles were complete d) Fully informed about the study and agreed to participate	a) Secondary polycythemia: erythrocytosis which are caused by congenital heart disease, chronic lung disease, methemoglobinemia, Wilms tumor, mild hypertension, etc
	b) Combined tumor, severe infection, or autoimmune disease, or ongoing hormones, immunosuppressive agents and other treatments that may affect complete blood cell counts
	c) Previous history of hyperlipidemia, or use drugs that may affect blood lipid parameters in nearly 2 months
	d) Combined with serious heart, liver, kidney, and other organs lesions that may die in a short period of time

### Methods

The PHR, NHR, MHR, LHR, and SIRI were calculated using the following formulae: PHR, platelet/HDL ratio; NHR, neutrophil/HDL ratio; MHR, monocyte/HDL ratio; LHR, lymphocyte/HDL ratio; and SIRI, monocyte × neutrophil-to-lymphocyte ratio.

The definition of PV-related thrombosis: arterial thrombosis includes cerebral infarction, myocardial infarction, and peripheral artery disease. Venous thrombosis includes pulmonary embolism, splanchnic vein thrombosis, and deep vein thrombosis. Thrombosis risk grouping includes (a) low-risk group, age ≤ 60 years and no history of thrombosis, and (b) high-risk group, age > 60 years or history of thrombosis. Thrombosis progression during follow-up includes (a) no history of thrombosis but thrombosis events occurred during follow-up and (b) at least one record history of thrombosis with progression in the same site or new thrombosis events in different sites. JAK2 mutation: presence of JAK2 V617F or JAK2 exon 12 mutation. Cardiovascular risk factors (CVF) include smoking, hypertension, and diabetes; hyperlipidemia was excluded.

The clinical data of the patients included gender, age, body mass index (BMI), history of thrombosis, JAK2 mutation, complete blood count, blood lipid data, and therapeutic drugs. The patients were followed up through inpatient medical records, outpatient medical records, and telephone calls. The last follow-up was until June 2023. Progression-free survival (PFS) time is defined as the time from the initial diagnosis until the occurrence of thrombotic progression events or the last follow-up.

### Statistical analysis

Statistical analyses were performed using SPSS version 27.0 software (SPSS, Inc., Chicago, IL, USA). Graphs were created using GraphPad Prism 9.0 (GraphPad Software). Continuous variables conformed to a normal distribution were described as means ± standard deviations (SDs) and were subsequently analyzed using the Student *t-*test, while those not normally distributed were expressed as the median and interquartile range (IQR) and then analyzed using the Mann–Whitney *U* test. Categorical variables are expressed as numbers and percentages of patients (*n*) % and analyzed using the chi-squared test and Fisher’s exact test. Cox hazard regression models were used for the analysis of impact factors and to calculate the hazard ratio (HR) and 95% confidence intervals (95% CI). The optimum cut-off value for identifying thrombotic progression in this sample was calculated using ROC curve analysis. The optimal cut-off value was determined by the maximum Youden index. Survival analyses were compared using the Kaplan–Meier method, and log-rank tests were used to compare the differences between groups. A bilateral *P* < 0.05 was considered statistically significant.

## Results

### Comparison of baseline clinical characteristics and laboratory indicators between the PV patients with different risk of thrombosis and control group

Table 2 summarizes the baseline clinical characteristics and laboratory parameters of PV patients with different risk of thrombosis and control group. A total of 148 PV patients (90 males and 58 females) with a median age of 57 years (49–66 years) at diagnosis were included. Patients were divided into low-risk group (42.6%) and high-risk group (57.4%) according to the risk of thrombosis. Compared to the low-risk group, the high-risk group had a higher percentage of history of thrombosis (58.8% vs. 0%, *P* < 0.001) and JAK2 mutation rate (85.9% vs. 66.7%, *P* = 0.005), and showed significantly increased levels of age (65 vs. 52 years, *P* < 0.001), white blood cells (10.7 vs. 8.5, *P* < 0.001), platelets (329 vs. 243, *P* < 0.001), neutrophils (6.8 vs. 5.4, *P* < 0.001), monocytes (0.42 vs. 0.34, *P* = 0.004), PHR (314.4 vs. 204.4, *P* < 0.001), NHR (6.32 vs. 4.89, *P* < 0.001), NLR (5.69 vs. 4.19, *P* < 0.001), MHR (0.39 vs. 0.30, *P* = 0.006), and SIRI (2.33 vs. 1.32, *P* < 0.001). There were no differences in gender, at least one cardiovascular risk factor, hemoglobin, lymphocytes, hematocrit, cholesterol, triglyceride, high-density lipoprotein, and LHR (*P* > 0.05). In addition, there was no difference in aspirin, phlebotomy, and interferon therapy between the two groups (*P* > 0.05). More PV patients in the high-risk group received hydroxyurea (72.9% vs. 52.4%, *P* = 0.01).

**Table 2:** Baseline characteristics of PV patients with different risk of thrombosis and control group

	PV			*P* value (low vs high)	Control (*n* = 148)	*P* value (PV vs control)
	Total (*n* = 148)	Low risk (*n* = 63)	High risk (*n* = 85)			
Gender, male, *n* (%)	90 (60.8%)	43 (68.3%)	47 (55.3%)	0.11	88 (59.5%)	0.81
Age, years, median (IQR)	57 (49–66)	52 (45–58)	65 (54–71)	< 0.001	59 (51–68)	0.2
BMI, kg/m^2^, mean ± SD	21.8 ± 2.7	22.4 ± 2.9	21.4 ± 2.4	0.03	22.2 ± 2.7	0.17
Previous thrombosis, *n* (%)	50 (33.8%)	0	50 (58.8%)	< 0.001	—	—
JAK2 mutation, *n* (%)	115 (77.7%)	42 (66.7%)	73 (85.9%)	0.005	—	—
At least one CV risk, *n* (%)	80 (54.1%)	36 (57.1%)	44 (51.8%)	0.52	47 (31.8%)	< 0.001
Laboratory data, median (IQR)						
White blood cells, × 10^9^/L	9.5 (8.3–10.9)	8.5 (7.5–9.2)	10.7 (9.2–11.9)	< 0.001	5.3 (4.6–6.2)	< 0.001
Hemoglobin (g/L)	189 (181–201)	189 (180–203)	189 (183–200)	0.64	133 (123–146)	< 0.001
Platelets, × 10^9^/L	289 (192–429)	243 (177–336)	329 (231–505)	< 0.001	203 (176–240)	< 0.001
Neutrophils, × 10^9^/L	6.2 (5.1–7.5)	5.4 (4.7–6.4)	6.8 (5.5–8.2)	< 0.001	3.2 (2.7–3.9)	< 0.001
Lymphocyte, × 10^9^/L	1.25 (1.08–1.57)	1.35 (1.12–1.67)	1.23 (1.07–1.52)	0.16	1.61 (1.33–2.04)	< 0.001
Monocyte, × 10^9^/L	0.37 (0.29–0.51)	0.34 (0.25–0.43)	0.42 (0.33–0.54)	0.004	0.36 (0.27–0.45)	0.07
Hematocrit, (%)	57.6 (54.5–61.4)	57.7 (54.3–62.2)	57.3 (55.2–60.9)	0.61	40.7 (38.3–44)	< 0.001
TC, mmol/L	3.85 (3.47–4.42)	4.01 (3.54–4.58)	3.73 (3.32–4.3)	0.08	4.42 (4.05–4.71)	< 0.001
TG, mmol/L	1.33 (1.09–1.55)	1.37 (1.18–1.56)	1.31 (1.05–1.55)	0.18	1.13 (0.91–1.45)	< 0.001
HDL-C, mmol/L	1.09 (0.89–1.09)	1.08 (0.95–1.27)	1.09 (1–1.23)	0.83	1.31 (1.07–1.31)	< 0.001
LDL-C, mmol/L	2.09 (1.71–2.68)	2.20 (1.89–2.99)	2.07 (1.67–2.52)	0.03	2.76 (2.25–3.63)	< 0.001
PHR, × 10^9^/mmol	261.6 (176–391)	204.4 (150–311)	314.4 (210–445)	< 0.001	158.9 (133–189)	< 0.001
MHR, × 10^9^/mmol	0.35 (0.25–0.46)	0.30 (0.23–0.41)	0.39 (0.29–0.47)	0.006	0.28 (0.2–0.35)	< 0.001
NHR, × 10^9^/mmol	5.49 (4.38–7.16)	4.89 (4.13–6.02)	6.32 (4.88–7.64)	< 0.001	2.51 (2.07–3.1)	< 0.001
NLR	4.86 (3.43–6.36)	4.19 (3.12–5.43)	5.69 (3.68–7.40)	< 0.001	1.93 (1.51–2.71)	< 0.001
LHR, × 10^9^/mmol	1.16 (0.92–1.45)	1.18 (0.92–1.6)	1.14 (0.92–1.39)	0.32	1.22 (0.98–1.65)	0.09
SIRI, 10^9^/L	1.81 (1.2–2.71)	1.32 (0.93–1.99)	2.33 (1.52–3.05)	< 0.001	0.63 (0.49–1)	< 0.001
Treatments, *n* (%)						
Aspirin	84 (56.8%)	31 (49.2%)	53 (62.4%)	0.11	—	—
Phlebotomy	43 (29.1%)	19 (30.2%)	24 (28.2%)	0.8	—	—
Hydroxyurea	95 (64.2%)	33 (52.4%)	62 (72.9%)	0.01	—	—
Interferon	98 (66.2%)	47 (74.6%)	51 (60%)	0.06	—	—

*PV* polycythemia vera, *IQR* interquartile range, *BMI* body mass index, *CV* cardiovascular, *TC* total cholesterol, *TG* triglyceride, *HDL-C* high-density lipoprotein cholesterol, *LDL-C* low-density lipoprotein cholesterol, *PHR* platelet/HDL-C ratio, *MHR* monocyte/HDL-C ratio, *NHR* neutrophil/HDL-C ratio, *NLR* neutrophil/lymphocyte ratio, *LHR* lymphocyte/HDL-C ratio, *SIRI* system inflammation response index, *P* < 0.05 (two-sided) was defined as statistically significant. Bold values indicate statistically significance.

Comparing the baseline clinical characteristics and laboratory parameters, WBC (9.5 vs. 5.3, *P* < 0.001), hemoglobin (189 vs. 133, *P* < 0.001), platelet (289 vs. 203, *P* < 0.001), neutrophils (6.2 vs. 3.2, *P* < 0.001), hematocrit (57.6% vs. 40.7%, *P* < 0.001), triglyceride (1.33 vs. 1.13, *P* < 0.001), PHR (261.6 vs. 158.9, *P* < 0.001), NHR (5.49 vs. 2.51, *P* < 0.001), NLR (4.86 vs. 1.93, *P* < 0.001), MHR (0.35 vs. 0.28, *P* < 0.001), and SIRI (1.81 vs. 0.63, *P* < 0.001) were significantly higher in the PV group than that in the healthy control group. Conversely, lymphocytes (1.25 vs. 1.61, *P* < 0.001), cholesterol (3.85 vs. 4.42, *P* < 0.001), high density lipoprotein (1.09 vs. 1.31, *P* < 0.001), and low density lipoprotein (2.09 vs. 2.76, *P* < 0.001) were significantly lower. In addition, there was no difference between the two groups in terms of gender, age, BMI, monocyte count, and LHR (*P* > 0.05).

**Fig. 2:**
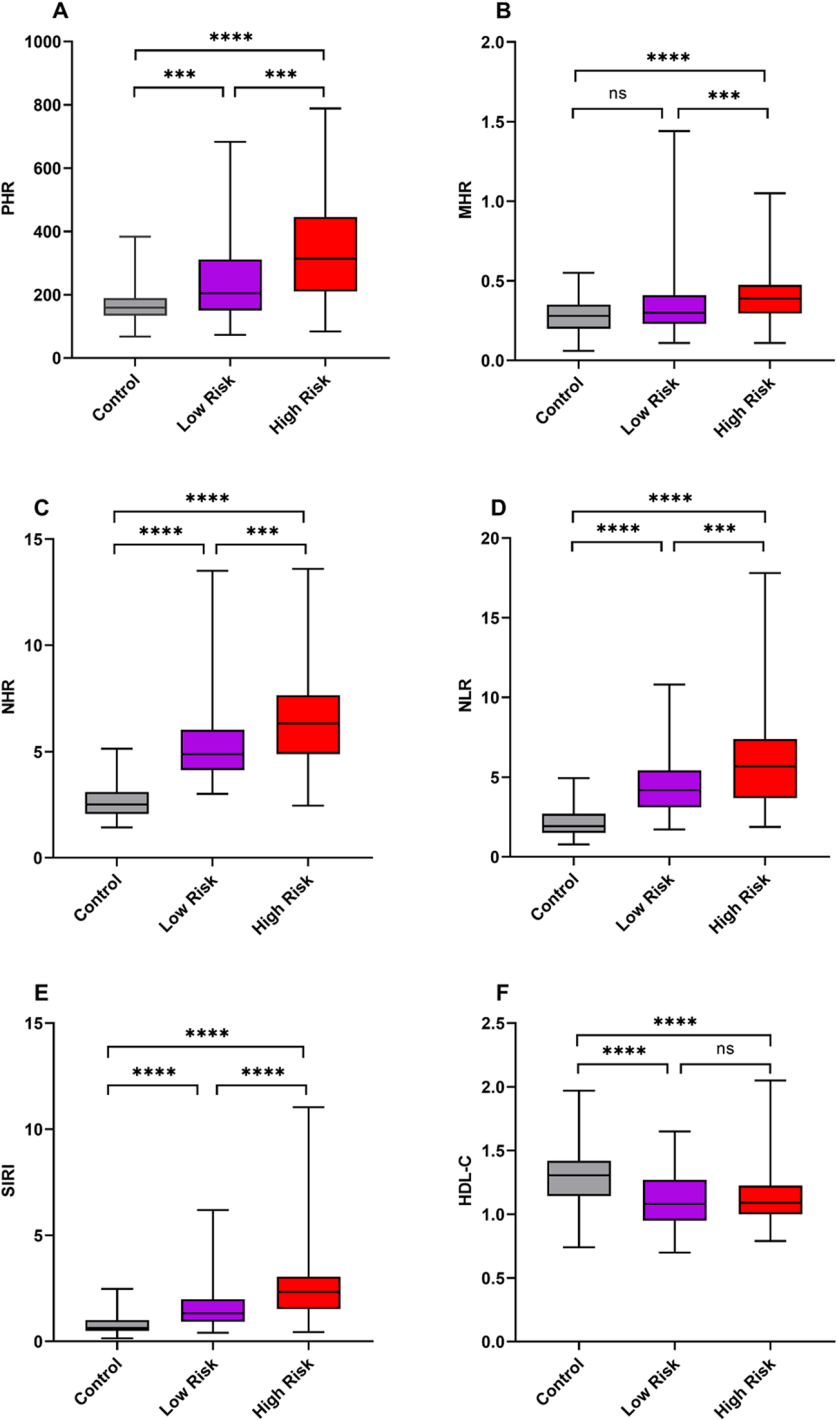
The levels of PHR, MHR, NHR, NLR, SIRI and HDL-C were compared between control group, low-risk group, and high-risk group (**A**–**F)**. On the box plots, central lines represent the median, the length of the box represents the interquartile range, and the lines extend to minimum and maximum values. ****P < 0.0001; ***P < 0.001; **P < 0.01; *P < 0.05. ns, no statistical significance

The levels of PHR, MHR, NHR, NLR, SIRI, and HDL-C of low-risk group, high-risk group, and control group are summarized in Fig. 2 (A–F). The box plots showed considerably higher levels of PHR, MHR, NHR, NLR, and SIRI than low-risk group and control group (*P* < 0.001). Compared to the control group, the low-risk group showed significantly higher levels of PHR, NHR, NLR, and SIRI (*P* < 0.001), while there was no difference in MHR (*P* > 0.05). The control group had significantly higher levels of HDL-C than those in the low-risk group and high-risk group (*P* < 0.0001), while low-risk group and high-risk group did not differ regarding HDL-C (*P* > 0.05).

### Types of 33 nonfatal thromboses during follow-up

During a median follow-up of 4.02 years, 33 thromboses (22.3%, 29 arterial and 4 venous) were recorded in 148 PV patients. Arterial thromboses were mostly cerebral arterial thrombosis, while venous thromboses were mostly deep vein thrombosis (Table 3).

**Table 3:** Types of 33 nonfatal thromboses during follow-up (median follow-up time, 4.02 years)

Nonfatal thrombosis	*N* (%)
Arterial	29 (87.9%)
Cerebral arterial thrombosis	24 (72.7%)
Myocardial infarction	1 (3%)
Peripheral arterial thrombosis	2 (6.1%)
Splenic artery thrombosis	2 (6.1%)
Venous	4 (12.1%)
Deep vein thrombosis	4 (12.1%)

In nonfatal thrombosis, no patients died from arterial or venous thrombotic events.

### Comparison of baseline clinical characteristics and laboratory indicators between the PV patients with or without progression of thrombosis

The baseline clinical characteristics and laboratory parameters of PV patients with or without progression of thrombosis are summarized in Table 4. A total of 148 PV patients were divided into thrombosis non-progression group (77.7%) and progression group (22.3%) according to whether thrombosis progression has occurred. Compared to the thrombosis non-progression group, thrombosis progression group had a higher percentage of history of thrombosis (51.5% vs. 28.7%, *P* = 0.015) and showed significantly increased levels of WBC (10.8 vs. 9.1, *P* < 0.001), platelet (364 vs. 266, *P* = 0.04), neutrophils (7.4 vs. 5.8, *P* < 0.001), PHR (345.5 vs. 237.0, *P* < 0.001), NHR (7.18 vs. 5.04, *P* < 0.001), NLR (6.36 vs. 4.53, *P* = 0.006), and SIRI (2.35 vs. 1.69, *P* = 0.02), while the levels of HDL-C (1.02 vs. 1.12, *P* < 0.001) significantly decreased. The two groups did not differ regarding gender, age, BMI, JAK2 mutation, at least one cardiovascular risk factor, hemoglobin, lymphocytes, monocytes, hematocrit, cholesterol, triglyceride, LDL-C, MHR, and LHR (*P* > 0.05).

**Table 4:** Baseline characteristics of PV patients grouped according to with or without progression of thrombosis

	No progression of thrombosis (*n* = 115)	Progression of thrombosis (*n* = 33)	*P* value
Gender, male, *n* (%)	71 (61.7%)	19 (57.6%)	0.67
Age, years, median (IQR)	58 (49–66)	54 (50–66)	0.59
BMI, kg/m^2^, mean ± SD	21.7 ± 2.6	22.3 ± 2.8	0.29
Previous thrombosis, n (%)	33 (28.7%)	17 (51.5%)	0.015
JAK2 mutation, n (%)	87 (75.7%)	28 (84.8%)	0.26
At least one CV risk, n (%)	61 (53%)	19 (57.6%)	0.65
Laboratory data, median (IQR)			
White blood cells, × 10^9^/L	9.1 (8.2–10.7)	10.8 (9.2–12.5)	< 0.001
Hemoglobin (g/L)	188 (180–200)	194 (186–205)	0.07
Platelets, × 10^9^/L	266 (183–426)	364 (252–485)	0.04
Neutrophils, × 10^9^/L	5.8 (4.9–7.1)	7.4 (6–9.8)	< 0.001
Lymphocyte, × 10^9^/L	1.26 (1.1–1.57)	1.21 (1.02–1.61)	0.9
Monocyte, × 10^9^/L	0.38 (0.28–0.5)	0.37 (0.29–0.52)	0.83
Hematocrit, (%)	57.3 (54.1–61.2)	58 (56.3–61.8)	0.23
TC, mmol/L	3.77 (3.48–4.4)	4.19 (3.14–4.51)	0.48
TG, mmol/L	1.32 (1.09–1.55)	1.37 (1.09–1.57)	0.45
HDL-C, mmol/L	1.12 (1.01–1.28)	1.02 (0.94–1.11)	< 0.001
LDL-C, mmol/L	2.09 (1.69–2.53)	2.17 (1.97–3)	0.14
PHR, × 10^9^/mmol	237 (164.7–337.2)	345.5 (246.2–468.3)	0.003
MHR, × 10^9^/mmol	0.34 (0.24–0.45)	0.38 (0.31–0.51)	0.15
NHR, × 10^9^/mmol	5.04 (4.2–6.44)	7.18 (6.06–9.03)	< 0.001
NLR	4.53 (3.40–5.95)	6.36 (3.91–8.51)	0.006
LHR, × 10^9^/mmol	1.14 (0.91–1.4)	1.29 (1–1.64)	0.07
SIRI, 10^9^/L	1.69 (1.13–2.42)	2.35 (1.57–3.18)	0.02
Treatments, *n* (%)			
Aspirin	65 (56.5%)	19 (57.6%)	0.91
Phlebotomy	38 (33%)	13 (39.4%)	0.5
Hydroxyurea	68 (59.1%)	24 (72.7%)	0.16
Interferon	75 (65.2%)	23 (69.7%)	0.63
Thrombosis risk, (%)			
Low risk	55 (47.8%)	8 (24.2%)	0.016
High risk	60 (52.2%)	25 (75.8%)	

*IQR* interquartile range, *BMI* body mass index, *CV* cardiovascular, *TC* total cholesterol, *TG* triglyceride, *HDL-C* high-density lipoprotein cholesterol, *LDL-C* low-density lipoprotein cholesterol, *PHR* platelet/HDL-C ratio, *MHR* monocyte/HDL-C ratio, *NHR* neutrophil/HDL-C ratio, *NLR* neutrophil/lymphocyte ratio, *LHR* lymphocyte/HDL-C ratio, *SIRI* system inflammation response index, *P* < 0.05 (two-sided) was defined as statistically significant. Bold values indicate statistically significance.

There was no difference between the two groups in terms of treatment with aspirin (57.6% vs. 56.5%, *P* = 0.91), phlebotomy (39.4% vs. 33%, *P* = 0.5), hydroxyurea (72.7% vs. 59.1%, *P* = 0.16), and interferon (69.7% vs. 65.2%, *P* = 0.16) therapy (*P* > 0.05). Thus, the impact of different therapy to PV patients with thrombosis progression can be ignored. In addition, the thrombosis progression group had a higher percentage of patients with high-risk than thrombosis non-progression group (75.8% vs. 52.2%, *P* = 0.016), which indicated that PV patients with high-risk of thrombosis showed an easier trend for thrombosis.

**Fig. 3:**
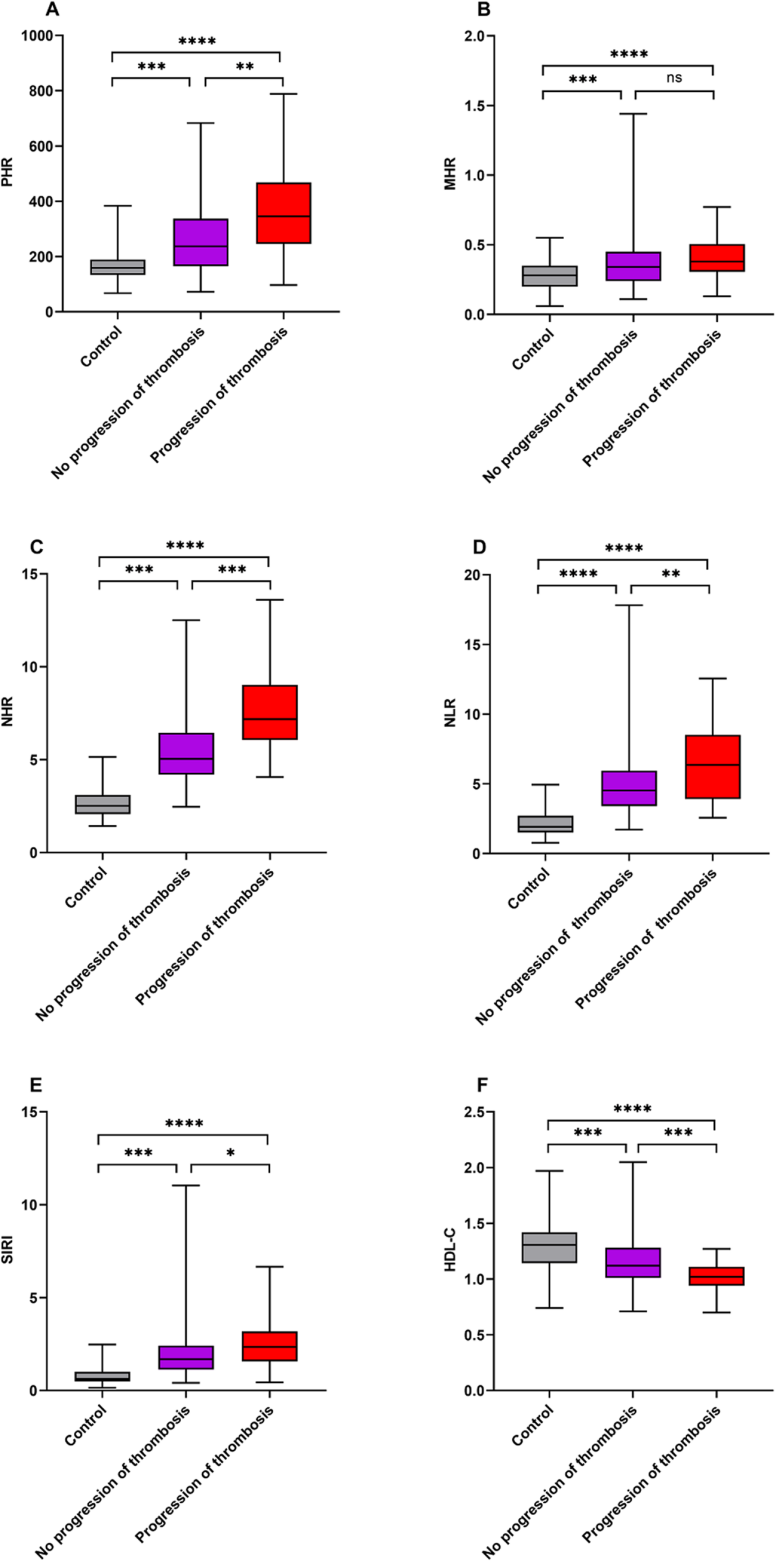
The levels of PHR, MHR, NHR, NLR, SIRI, and HDL-C were compared between control group, thrombosis non-progression group, and thrombosis progression group (**A**–**F)**. On the box plots, central lines represent the median, the length of the box represents the interquartile range and the lines extend to minimum and maximum values. ****P < 0.0001; ***P < 0.001; **P < 0.01; *P < 0.05. ns, no statistical significance

Figure 3 (A–F) summarizes the levels of PHR, MHR, NHR, NLR, SIRI, and HDL-C of thrombosis non-progression group, thrombosis progression group, and control group. The box-plot indicated that thrombosis progression group had higher levels of PHR, NHR, NLR, and SIRI than thrombosis non-progression group and control group (*P* < 0.05), while the levels of HDL-C showed significantly decreased (*P* < 0.001). There was no difference in the levels of MHR between thrombosis non-progression group and thrombosis progression group (*P* > 0.05), but both were significantly higher than those in the control group (*P* < 0.001). Compared to the control group, thrombosis non-progression group showed significantly increased levels of PHR, NHR, NLR, and SIRI (*P* < 0.001), while the levels of HDL-C showed significantly decreased (*P* < 0.001).

### Diagnostic performance of different inflammatory indexes for PV patients with progression of thrombosis during follow-up

**Fig. 4:**
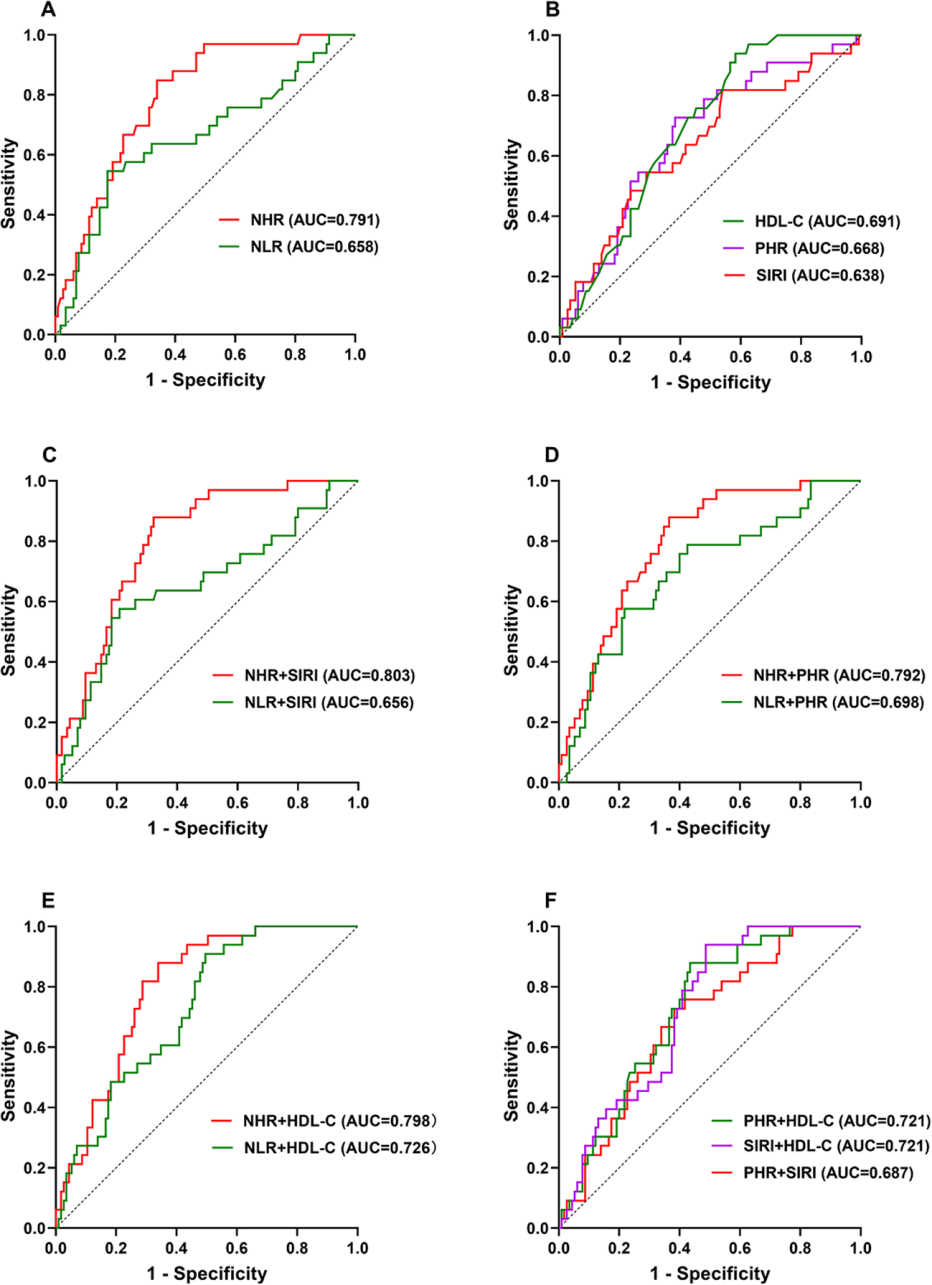
ROC curve analysis of the ability of NHR, NLR, HDL-C, PHR, and SIRI to predict PV patients with thrombosis progression during follow-up (**A**, **B**). ROC curve analysis of the ability of combined model of NHR, NLR, HDL-C, PHR, and SIRI to predict PV patients with thrombosis progression during follow-up (**C**–**F**). AUC, the area under the ROC curve

ROC curve analysis was used to evaluate the ability of NHR, NLR, HDL-C, PHR, and SIRI to identify PV patients with progression of thrombosis during follow-up. The results of the ROC curve analysis showed that NHR (AUC = 0.791, 95% CI, 0.713–0.869, *P* < 0.0001, cut-off 5.82) had an AUC greater than 0.7 which exhibited a high discriminating value for PV patients with thrombosis progression during follow-up. Besides, the other indexes with AUCs greater than 0.6 were the HDL-C (AUC = 0.691, 95% CI, 0.603–0.779, *P* = 0.0009, cut-off 1.22), PHR (AUC = 0.668, 95% CI, 0.567–0.770, *P* = 0.0032, cut-off 280.4), NLR (AUC = 0.658, 95% CI, 0.545–0.771, *P* = 0.006, cut-off 6.295), and SIRI (AUC = 0.638, 95% CI, 0.529–0.748, *P* = 0.0156, cut-off 1.53); their diagnostic efficacy is not as high as NHR. The data are presented in Fig. 4 A–B.

Additionally, the combination model of NHR, NLR, HDL-C, PHR, and SIRI showed a higher diagnostic efficacy for PV patients with progression of thrombosis during follow-up. NHR + SIRI (AUC = 0.803, 95% CI, 0.728–0.878, *P* < 0.0001), NHR + HDL-C (AUC = 0.798, 95% CI, 0.725–0.871, *P* < 0.0001), NHR + PHR (AUC = 0.792, 95% CI, 0.715–0.870, *P* < 0.0001), NLR + HDL-C (AUC = 0.726, 95% CI, 0.640–0.813, *P* < 0.0001), SIRI + HDL-C (AUC = 0.721, 95% CI, 0.637–0.806, *P* = 0.0001), and PHR + HDL-C (AUC = 0.721, 95% CI, 0.634–0.807, *P* = 0.0001) all had an AUC > 0.7 which showed a higher diagnostic value than single index. The NLR + PHR (AUC = 0.698, 95% CI, 0.596–0.800, *P* = 0.0005), PHR + SIRI (AUC = 0.687, 95% CI, 0.593–0.782, *P* = 0.0011), and NLR + SIRI (AUC = 0.656, 95% CI, 0.543–0.769, *P* = 0.006) had an AUC > 0.6 which showed an ordinary diagnostic value. The data are presented in Fig. 4 C–F.

The above results indicated that compared to the NLR, NHR has a higher diagnostic efficacy for early identification of thrombosis progression during follow-up. In addition, the combined model of NHR, NLR, HDL-C, PHR, and SIRI showed a higher diagnostic value than the separate models.

### Univariate and multivariate Cox hazards analysis of the risk factors for PV patients with progression of thrombosis during follow-up

ROC curve was used to calculated the optimum cut-off values of WBC, PLT, NHR, NLR, MHR, and LHR to identify the progression of thrombosis in patients with PV during the follow-up period, and all patients were divided into two groups according to the results (Table 5). The optimum cut-off values of WBC, PLT, NHR, NLR, MHR, and LHR were 9.15 × 10^9^/L, 307.5 × 10^9^/L, 5.82 × 10^9^/mmol, 6.295, 0.295 × 10^9^/mmol, and 1.41 × 10^9^/mmol. Univariate Cox regression analysis showed that WBC (HR = 2.595, 95% CI, 1.125–5.988, *P* = 0.025), PLT (HR = 2.652, 95% CI, 1.275–5.518, *P* = 0.009), NHR (HR = 6.751, 95% CI, 2.603–17.509, *P* < 0.001), NLR (HR = 4.074, 95% CI, 2.046–8.114, *P* < 0.001), MHR (HR = 3.23, 95% CI, 1.326–7.865, *P* = 0.01), and LHR (HR = 2.156, 95% CI, 1.07–4.341, *P* = 0.032) were risk factors for the progression-free survival (PFS) of PV patients. These factors were included in the multivariate Cox regression analysis. After adjustment for confounding variables, NHR (HR = 3.955, 95% CI, 1.272–12.296, *P* = 0.017), NLR (HR = 3.934, 95% CI, 1.574–9.831, *P* = 0.003), MHR (HR = 2.647, 95% CI, 1.012–6.925, *P* = 0.047), and LHR (HR = 3.044, 95% CI, 1.360–6.810, *P* = 0.007) were still significantly correlated with thrombosis progression in PV patients (*P* < 0.05). However, the association of PFS with WBC and PLT was attenuated.

**Table 5:** Univariate and multivariate Cox hazards analysis of the influencing factors for PV patients with progression of thrombosis

Variable	Univariate analysis (PFS)		Multivariate analysis (PFS)	
	HR (95% CI)	*P* value	HR (95% CI)	*P* value
Gender (male vs. female)	0.989 (0.495–1.976)	0.974	**—**	**—**
Age (≤ 60 y vs. > 60 y)	1.264 (0.629–2.542)	0.511	**—**	**—**
Previous thrombosis (No vs. Yes)	1.838 (0.926–3.647)	0.082	**—**	**—**
JAK2 mutation (No vs. Yes)	1.619 (0.622–4.209)	0.323	**—**	**—**
WBC (< 9.15 × 10^9^/L vs. ≥ 9.15 × 10^9^/L)	2.595 (1.125–5.988)	**0.025**	0.464 (0.148–1.457)	0.189
PLT (< 307.5 × 10^9^/L vs. ≥ 307.5 × 10^9^/L)	2.652 (1.275–5.518)	**0.009**	1.802 (0.815–3.982)	0.146
NHR (< 5.82 × 10^9^/mmol vs. ≥ 5.82 × 10^9^/mmol)	6.751 (2.603–17.509)	< **0.001**	3.955 (1.272–12.296)	**0.017**
NLR (< 6.295 vs. ≥ 6.295)	4.074 (2.046–8.114)	< **0.001**	3.934 (1.574–9.831)	**0.003**
MHR (< 0.295 × 10^9^/mmol vs. ≥ 0.295 × 10^9^/mmol)	3.23 (1.326–7.865)	**0.01**	2.647 (1.012–6.925)	**0.047**
LHR (< 1.41 × 10^9^/mmol vs. ≥ 1.41 × 10^9^/mmol)	2.156 (1.07–4.341)	**0.032**	3.044 (1.360–6.810)	**0.007**

*PFS* progression-free survival, *HR* hazard ratio, *95% CI* 95% confidence interval, *NHR* neutrophil/HDL-C ratio, *NLR* neutrophil/lymphocyte ratio, *MHR* monocyte/HDL-C ratio, *LHR* lymphocyte/HDL-C ratio, *P* < 0.05 (two-sided) was defined as statistically significant. Bold values indicate statistically significance.

### The Kaplan–Meier survival curves of categorized patients according to the optimal cut-off values of NHR, NLR, PHR, MHR, LHR, and SIRI

**Fig. 5:**
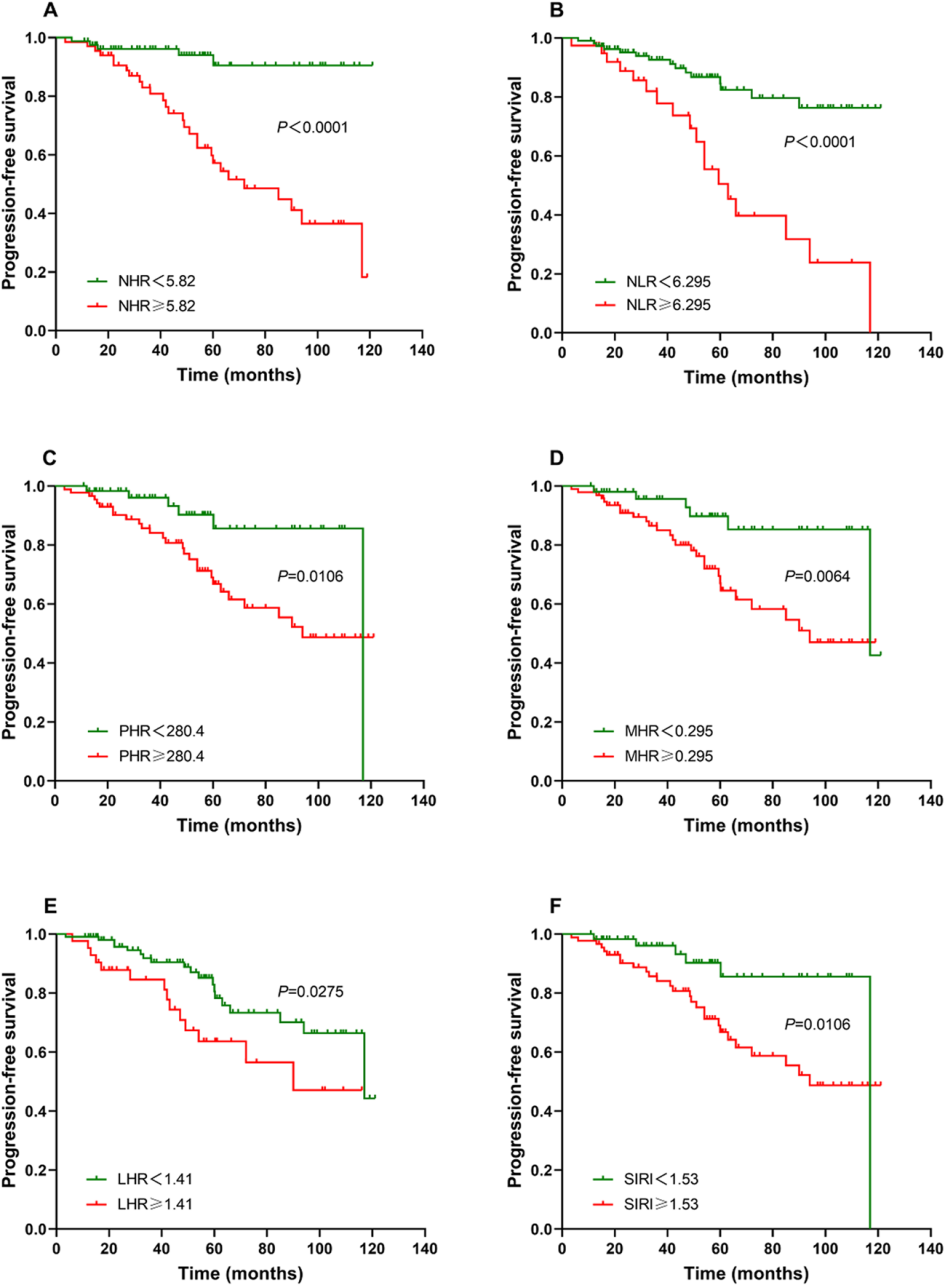
The Kaplan–Meier survival curves based on the NHR, NLR, PHR, MHR, LHR, and SIRI optimal cut-off values. Kaplan–Meier curves of categorized patients according to the optimal cut-off values of NHR (**A**), NLR (**B**), PHR (**C**), MHR (**D**), LHR (**E**), and SIRI (**F**). NHR, neutrophil/HDL-C ratio. NLR, neutrophil/lymphocyte ratio. PHR, platelet/HDL-C ratio. MHR, monocyte/HDL-C ratio. LHR, lymphocyte/HDL-C ratio. SIRI, system inflammation response index. *p* < 0.05 (two-sided) was defined as statistically significant

At the end of follow-up, a total of 33 (22.3%) PV patients (19 men, 14 women) occurred thrombosis progression. The optimal cut-off values of NHR (cut-off 5.82, *P* < 0.0001), NLR (cut-off 6.295, *P* < 0.0001), PHR (cut-off 280.4, *P* = 0.0032), MHR (cut-off 0.295, *P* = 0.146), LHR (cut-off 1.41, *P* = 0.066), and SIRI (cut-off 1.53, *P* = 0.0156) were calculated using ROC curve analysis. The Kaplan–Meier survival curves of categorized patients according to the optimal cut-off values of NHR, NLR, PHR, MHR, LHR, and SIRI are presented in Fig. 5. The results showed that NHR ≥ 5.82 × 10^9^/mmol, NLR ≥ 6.295, PHR ≥ 280.4 × 10^9^/mmol, MHR ≥ 0.295 × 10^9^/mmol, LHR ≥ 1.41 × 10^9^/mmol, and SIRI ≥ 1.53 × 10^9^/L were the risk factors for PFS in PV patients. Compared to the high NHR group (NHR ≥ 5.82), low NHR group (NHR < 5.82) had a significant longer median PFS (mPFS) (HR = 0.15, 95% CI, 0.075–0.297, *P* < 0.0001) (Fig. 5A). Patients in the low NLR group (NLR < 6.295) had a significant longer mPFS than those in the high NLR group (NLR ≥ 6.295) (HR = 0.249, 95% CI, 0.111–0.559, *P* < 0.0001) (Fig. 5B). In additional, patients in the low PHR group (PHR < 280.4) had a 23-month longer mPFS than those in the high PHR group (PHR ≥ 280.4) (HR = 0.334, 95% CI, 0.167–0.671, *P* = 0.0106) (Fig. 5C). Patients in the low MHR group (MHR < 0.295) also had a 23-month longer mPFS than those in the high MHR group (MHR ≥ 0.295) (HR = 0.315, 95% CI, 0.157–0.629, *P* = 0.0064) (Fig. 5D). Patients in the low LHR group (LHR < 1.41) had a 27-month longer mPFS than those in the high LHR group (LHR ≥ 1.41) (HR = 0.471, 95% CI, 0.216–1.028, *P* = 0.0275) (Fig. 5E). Compared to the high SIRI group (SIRI ≥ 1.53), patients in the low SIRI group (SIRI < 1.53) had a 23-month longer mPFS (HR = 0.334, 95% CI, 0.167–0.671, *P* = 0.0106) (Fig. 5F).

## Discussion

The Mayo Clinic had found that after a median follow-up of 665 patients with PV for 20 years, thrombotic events occurred in about 26% of the patients, indicating a high risk of thrombosis [3]. The results of another study in 1545 PV patients with a median follow-up time of approximately 7 years showed that thrombotic complications were another common cause of death in PV patients after leukemic transformation and secondary malignant tumors [18]. To date, no therapeutic strategy has been shown to be effective in reducing the risk of PV transforming into myelofibrosis or leukemia, and the main goal of current treatment is to prevent thrombotic events. The existing PV thrombosis risk grouping is divided into low-risk group and high-risk group based on age and/or previous history of PV-related thrombosis, but early predictors for follow-up thrombosis progression are still lacking [16]. Recent studies have identified that PHR, NHR, MHR, LHR, and SIRI are novel inflammatory biomarkers and have significant clinical value due to their easy access [19]. Nevertheless, there is no evidence of an association between these inflammatory parameters and PV patients with thrombosis progression. Based on the bidirectional effects of serologic inflammatory markers and HDL on thrombosis, this study confirmed for the first time that inflammation and abnormal lipid metabolism are associated with the high risk of thrombosis in PV patients. The novel inflammatory markers PHR, NHR, MHR, LHR, and SIRI could be used as new predictors for PV patients with thrombosis progression.

Previous studies have shown that serologic inflammatory markers and high-density lipoproteins are closely associated with thrombosis. Platelets can exert proinflammatory functions by inducing neutrophil extracellular trap formation, enhancing leucocyte recruitment, and secreting granular contents such as high mobility group protein B1. Then, these platelets participate in arterial and venous thrombosis by triggering coagulation through intrinsic pathways [20]. Activated neutrophils are key effectors of both arterial and venous thrombosis and can be targeted through immunoregulatory nanoparticles [21]. In addition, high monocyte counts (> 0.6 × 10^9^/L) were independently associated with an increased risk of deep vein thrombosis (DVT) in older patients with hip fracture (OR = 1.705, 95% CI, 1.121–2.593, *P* = 0.013) [22]. On the contrary, HDL-C has anti-inflammatory, anti-oxidative and anti-thrombotic effects, and the levels of HDL-C are significantly negatively correlated with the risk of development of retinal vein occlusion [23]. Our findings corroborated the above views and showed that the novel inflammatory markers PHR, NHR, MHR, LHR, and SIRI were closely related to thrombosis. In additional, the high-risk group had a higher incidence of thrombosis progression than the low-risk group (29.4% vs. 12.7%, *P* = 0.016), the finding that is consistent with that of a retrospective study in the USA [24]. Patients with preexisting hyperlipidemia or use of lipid-lowering medications that may affect blood lipid parameters in nearly 2 months were excluded, and therefore the effect of hyperlipidemia and medications on lipid parameters in the study population could be ignored.

There was no difference between the thrombosis non-progression and progression group in terms of treatment with aspirin, phlebotomy, hydroxyurea, and interferon, so that the impact of treatment regimen to PV patients with thrombosis progression can be ignored. Box plots (Fig. 3A–F) indicated that novel inflammatory markers PHR, NHR, NLR, and SIRI were closely related to PV patients with thrombosis progression and that HDL-C levels were significantly and negatively correlated with the risk of thrombosis. This is similar to the conclusions drawn from previous studies, such as PHR, NHR, NLR, and SIRI were respectively associated with immediate postoperative deep vein thrombosis in patients with open wedge high tibial osteotomy. Patients with ST-segment elevation myocardial infarction undergoing primary percutaneous coronary intervention developed stent thrombosis, venous thromboembolism after total knee replacement. Patients with aneurysmal subarachnoid hemorrhage after endovascular treatment developed DVT [25–28]. Therefore, we draw the conclusion that PHR, NHR, NLR, and SIRI have a high predictive value for PV patients with thrombosis progression.

ROC curve analysis indicated that NHR, HDL-C, PHR, NLR, and SIRI had high diagnostic efficacy for identifying PV patients with thrombosis progression. The levels of MHR and LHR did not differ between thrombosis non-progression group and thrombosis progression group so we did not include them in the ROC curve for analysis. Previous studies had indicated that patients with PV showed a higher level of NLR than the normal population, and NLR may not only be used as a novel predictor of venous thrombosis in PV, but also may become a new secondary criterion for diagnosis of PV [5, 29, 30]. In addition, PLR may represent a cheap and rapidly available biomarker for the diagnosis of PV [31]. Here we firstly confirmed that the levels of PHR, NHR, MHR, and SIRI in PV patients were significantly higher than those in the normal population. PHR, NHR, and SIRI may be new predictors for PV patients with thrombosis progression which also have certain reference value for the diagnosis of PV. NHR has the highest value in predicting thrombosis in PV patients and is superior to NLR which was reported previously (AUC = 0.791 vs. 0.658).

Cox regression analysis showed that WBC, PLT, NHR, NLR, MHR, and LHR were independent risk factors for PV patients with thrombosis progression. Consistent with the results of meta-analysis by Carobbio et al., WBC is an independent risk factor for PV patients with thrombosis [32]. Gender, age, history of thrombosis, and JAK2 mutation had no correlation with progression of thrombosis (*P* > 0.05). Previous studies had reported that JAK2V617F variant allele frequency and history of thrombosis were independent predictors of thrombosis in PV patients [33, 34]. However, we did not get the same results, and the most likely reason is that our study had an insufficient sample size and that we did not further investigate the correlation of JAK2V617F variant allele frequency with thrombosis in PV patients. Kaplan–Meier survival curves indicated that NHR, NLR, PHR, MHR, LHR, and SIRI were risk factors for achieving PFS in PV patients; we will follow up all patients for a long time and research the association between the above indicators and the overall survival of PV patients.

This retrospective study also has some limitations. First, the small sample size of this study did not confirm that JAK2 mutation and history of previous thrombosis were independent predictors of future thrombotic events in PV patients. In addition, only 77.7% of PV patients were positive for the JAK2 mutation gene, and another 22.3% were diagnosed by minor criterion. Therefore, we did not further analyze the correlation between JAK2V617F variant allele frequency and thrombosis in PV patients. Later, we can investigate the above questions based on larger sample size and research on the effect of JAK2V617F mutation allele frequency on the survival outcome of PV patients. Second, the median follow-up time of PV patients was 4 years, and only 22.3% of patients showed thrombosis progression. Thus, the median PFS time of high NHR group (NHR ≥ 5.82) and high NLR group (NLR ≥ 6.295) could not be derived. We will continue to follow up with all the PV patients with the aim of deriving more valuable findings. Third, although there was no difference in the therapy of aspirin, phlebotomy, and interferon in patients with different thrombotic risks (*P* > 0.05), more patients in the high-risk group were treated with hydroxyurea (*P* = 0.01), so the possible effect of hydroxyurea therapy as a confounding factor may be overlooked. Fourth, the novel inflammatory indicators were not dynamically monitored. Therefore, whether their changes are related to PV patients with thrombosis progression remains unknown. Further prospective studies are required to analyze whether the above indicators increase the risk of thrombosis in PV patients.

In conclusion, the study confirms for the first time that inflammation and abnormal lipid metabolism were closely related to the high risk of thrombosis in PV patients. The novel inflammatory markers PHR, NHR, MHR, LHR, and SIRI could be used as new predictors for PV patients with thrombosis progression. Additionally, NHR has the highest value in predicting thrombosis in PV patients and is superior to NLR which has been previously reported.

## Data Availability

The data sets generated and analyzed during the current study are available from the corresponding authors upon reason-able request.

## References

[1] Srour SA, Devesa SS, Morton LM, Check DP, Curtis RE, Linet MS, Dores GM (2016). Incidence and patient survival of myeloproliferative neoplasms and myelodysplastic/myeloproliferative neoplasms in the United States, 2001–12. Br J Haematol.

[2] Tefferi A, Guglielmelli P, Larson DR, Finke C, Wassie EA, Pieri L, Gangat N, Fjerza R, Belachew AA, Lasho TL, Ketterling RP, Hanson CA, Rambaldi A, Finazzi G, Thiele J, Barbui T, Pardanani A, Vannucchi AM (2014). Long-term survival and blast transformation in molecularly annotated essential thrombocythemia, polycythemia vera, and myelofibrosis. Blood.

[3] Szuber N, Mudireddy M, Nicolosi M, Penna D, Vallapureddy RR, Lasho TL, Finke C, Begna KH, Elliott MA, Hook CC, Wolanskyj AP, Patnaik MM, Hanson CA, Ketterling RP, Sirhan S, Pardanani A, Gangat N, Busque L, Tefferi A (2019). 3023 Mayo Clinic patients with myeloproliferative neoplasms: risk-stratified comparison of survival and outcomes data among disease subgroups. Mayo Clin Proc.

[4] Cerquozzi S, Barraco D, Lasho T, Finke C, Hanson CA, Ketterling RP, Pardanani A, Gangat N, Tefferi A (2017). Risk factors for arterial versus venous thrombosis in polycythemia vera: a single center experience in 587 patients. Blood Cancer J.

[5] Carobbio A, Vannucchi AM, De Stefano V, Masciulli A, Guglielmelli P, Loscocco GG, Ramundo F, Rossi E, Kanthi Y, Tefferi A, Barbui T (2022). Neutrophil-to-lymphocyte ratio is a novel predictor of venous thrombosis in polycythemia vera. Blood Cancer J.

[6] Wang Z, Liu W, Wang D, Yang E, Li Y, Li Y, Sun Y, Wang M, Lv Y, Hu X (2022). TET2 mutation may be more valuable in predicting thrombosis in ET patients compared to pv patients: a preliminary report. J Clin Med.

[7] Huang Y, Ge H, Wang X, Zhang X (2022). Association between blood lipid levels and lower extremity deep venous thrombosis: a population-based cohort study. Clin Appl Thromb Hemost.

[8] Podoltsev NA, Wang R, Shallis RM, Stempel JM, Di M, Neparidze N, Zeidan AM, Huntington SF, Giri S, Hull SC, Gore SD, Ma X (2023). Statin use, survival and incidence of thrombosis among older patients with polycythemia vera and essential thrombocythemia. Cancer Med.

[9] Huang JB, Chen YS, Ji HY, Xie WM, Jiang J, Ran LS, Zhang CT, Quan XQ (2020). Neutrophil to high-density lipoprotein ratio has a superior prognostic value in elderly patients with acute myocardial infarction: a comparison study. Lipids Health Dis.

[10] Han F, Sheng N, Sheng C, Meng J (2023). The diagnostic and prognostic value of haematologic parameters in multiple myeloma patients. Hematology.

[11] Liu Z, Fan Q, Wu S, Wan Y, Lei Y (2021). Compared with the monocyte to high-density lipoprotein ratio (MHR) and the neutrophil to lymphocyte ratio (NLR), the neutrophil to high-density lipoprotein ratio (NHR) is more valuable for assessing the inflammatory process in Parkinson's disease. Lipids Health Dis.

[12] Yu S, Guo X, Li G, Yang H, Zheng L, Sun Y (2021). Lymphocyte to high-density lipoprotein ratio but not platelet to lymphocyte ratio effectively predicts metabolic syndrome among subjects from rural china. Front Cardiovasc Med.

[13] Jialal I, Jialal G, Adams HB (2021). The platelet to high density lipoprotein-cholesterol ratio is a valid biomarker of nascent metabolic syndrome. Diabetes Metab Res Rev.

[14] Han K, Shi D, Yang L, Wang Z, Li Y, Gao F, Liu Y, Ma X, Zhou Y (2022). Prognostic value of systemic inflammatory response index in patients with acute coronary syndrome undergoing percutaneous coronary intervention. Ann Med.

[15] Guglielmelli P, Vannucchi AM (2020). Current management strategies for polycythemia vera and essential thrombocythemia. Blood Rev.

[16] Tefferi A, Barbui T (2023). Polycythemia vera: 2024 update on diagnosis, risk-stratification, and management. Am J Hematol.

[17] Arber DA, Orazi A, Hasserjian R, Thiele J, Borowitz MJ, Le BMM, Bloomfield CD, Cazzola M, Vardiman JW (2016). The 2016 revision to the World Health Organization classification of myeloid neoplasms and acute leukemia. Blood.

[18] Tefferi A, Rumi E, Finazzi G, Gisslinger H, Vannucchi AM, Rodeghiero F, Randi ML, Vaidya R, Cazzola M, Rambaldi A, Gisslinger B, Pieri L, Ruggeri M, Bertozzi I, Sulai NH, Casetti I, Carobbio A, Jeryczynski G, Larson DR, Müllauer L, Pardanani A, Thiele J, Passamonti F, Barbui T (2013). Survival and prognosis among 1545 patients with contemporary polycythemia vera: an international study. Leukemia.

[19] Song Y, Zhao Y, Shu Y, Zhang L, Cheng W, Wang L, Shu M, Xue B, Wang R, Feng Z, Yin Y, Yu F, Jin S (2023). Combination model of neutrophil to high-density lipoprotein ratio and system inflammation response index is more valuable for predicting peripheral arterial disease in type 2 diabetic patients: a cross-sectional study. Front Endocrinol.

[20] Stevens H, McFadyen JD (2019). Platelets as central actors in thrombosis-reprising an old role and defining a new character. Semin Thromb Hemost.

[21] Nayak L, Sweet DR, Thomas A, Lapping SD, Kalikasingh K, Madera A, Vinayachandran V, Padmanabhan R, Vasudevan NT, Myers JT, Huang AY, Schmaier A, Mackman N, Liao X, Maiseyeu A, Jain MK (2022). A targetable pathway in neutrophils mitigates both arterial and venous thrombosis. Sci Transl Med.

[22] Wang Z, Zhou Q, Liu H, Zhang J, Zhu Z, Wu J, Chen X, Liu Y (2022). Association between monocyte count and preoperative deep venous thrombosis in older patients with hip fracture: a retrospective study. Clin Appl Thromb Hemost.

[23] Kim J, Lim Dong H, Han K, Kang SW, Ham D, Kim SJ, Chung TY (2019). Retinal vein occlusion is associated with low blood high-density lipoprotein cholesterol: a nationwide cohort study. Am J Ophthalmol.

[24] Verstovsek S, Pemmaraju N, Reaven NL, Funk SE, Woody T, Valone F (2023). Real-world treatments and thrombotic events in polycythemia vera patients in the USA. Ann Hematol.

[25] Guo H, Wang T, Li C, Yu J, Zhu R, Wang M, Zhu Y, Wang J (2023) Development and validation of a nomogram for predicting the risk of immediate postoperative deep vein thrombosis after open wedge high tibial osteotomy. Knee Surg Sports Traumatol Arthrosc: Adv Online Publ10.1007/s00167-023-07488-8 10.1007/s00167-023-07488-8 37378681

[26] Chen Y, Jiang D, Tao H, Ge P, Duan Q (2022). Neutrophils to high-density lipoprotein cholesterol ratio as a new prognostic marker in patients with ST-segment elevation myocardial infarction undergoing primary percutaneous coronary intervention: a retrospective study. BMC Cardiovasc Disord.

[27] Seo WW, Park MS, Kim SE (2021). Neutrophil-lymphocyte ratio as a predictor of venous thromboembolism after total knee replacement. J Knee Surg.

[28] Qin Y, Zhang B, Zhao S, Wang W, Dong S, Miao Y, Zhao S, Liu L, Shen KT, Wu Z, Kang J (2023). Association between higher systemic immune inflammation index (SII) and deep vein thrombosis (DVT) in patients with aneurysmal subarachnoid hemorrhage (aSAH) after endovascular treatment. Neurosurg Rev.

[29] Kwon SS, Yoon SY, Jeong SY, Lee MY, Kim KH, Lee N, Won JH (2022). Neutrophil-lymphocyte ratio and carotid plaque burden in patients with essential thrombocythemia and polycythemia vera. Nutr Metab Cardiovasc Dis.

[30] Kim MJ, Kwon SS, Ji YS, Lee MY, Kim KH, Lee N, Park SK, Won JH, Yoon SY (2023). Neutrophil-to-lymphocyte ratio and platelet-to-lymphocyte ratio as new possible minor criteria for diagnosis of polycythemia vera.

[31] Krečak I, Holik H, Morić PM, Zekanović I, Coha B, Gverić KV, Lucijanić M (2022). High platelet-to-lymphocyte ratio may differentiate polycythemia vera from secondary polycythemia. Wien Klin Wochenschr.

[32] Carobbio A, Ferrari A, Masciulli A, Ghirardi A, Barosi G, Barbui T (2019). Leukocytosis and thrombosis in essential thrombocythemia and polycythemia vera: a systematic review and meta-analysis. Blood Adv.

[33] Moliterno AR, Kaizer H, Reeves BN (2023). JAK2 V617F allele burden in polycythemia vera: burden of proof. Blood.

[34] Guglielmelli P, Loscocco GG, Mannarelli C, Rossi E, Mannelli F, Ramundo F, Coltro G, Betti S, Maccari C, Ceglie S, Chiusolo P, Paoli C, Barbui T, Tefferi A, De SV (2021). JAK2V617F variant allele frequency >50% identifies patients with polycythemia vera at high risk for venous thrombosis. Blood Cancer J.

